# Electrospun Enzymatic Hydrolysis Lignin-Based Carbon Nanofibers as Binder-Free Supercapacitor Electrodes with High Performance

**DOI:** 10.3390/polym10121306

**Published:** 2018-11-26

**Authors:** Xiang Wang, Wei Zhang, Minzhi Chen, Xiaoyan Zhou

**Affiliations:** College of Wood Science &Technology, Nanjing Forestry University, 159, Longpan Road, Nanjing 210000, China; wxwoodscience@163.com (X.W.); 18362928689@163.com (W.Z.); chenminzhi@njfu.edu.cn (M.C.)

**Keywords:** EHL, carbon fiber, electrospinning, electrochemical performance

## Abstract

Carbon nanofibers consisting of Poly(acrylonitrile) (PAN) and enzymatic hydrolysis lignin (EHL) were prepared in the present study by electrospinning followed by stabilization in air and carbonization in N_2_ environment. The morphology and structure of the electrospun carbon nanofibers were characterized by Scanning Electron Microscopy (SEM), Brunauer-Emmett-Teller (BET), Roman, and the electrochemical performances were then evaluated by cyclic voltammetry (CV), galvanostatic charge-discharge (GCD), and electrochemical impedance spectroscopy (EIS)methods. When the amount of EHL was 60 wt. %, the as-prepared nanofibers have the smallest average diameter of 172 nm and the largest BET specific surface area of 675 m^2^/g without activating treatment. The carbon nanofiber electrode showed excellent specific capacitance of 216.8 F/g at the current density of 1 A/g, maintaining 88.8% capacitance retention after 2000 cycles. Moreover, the carbon nanofiber electrode containing 60 wt. % exhibited a smaller time constant (0.5 s) in comparison to that of carbon nanofibers in literatures. These findings suggest the potential use of EHL could be a practical as a sustainable alternative for PAN in carbon electrode manufacturing.

## 1. Introduction

With increasingly scarce of fossil fuels, the development of new sustainable energy storage device is necessary, such as Li-ion batteries, fuel cells, solar cells, and supercapacitors. Amongst these energy storage devices, the supercapacitors have attracted extensive attention due to large capacity, ultrahigh power density, exceptional cycle life, a wide range of operating temperatures, etc. [1,2], and hold great potential for a wide range of applications, such as hybrid electric vehicles and electronic devices. In general, the supercapacitors can be classified into electrochemical double-layer capacitors (EDLCs) and pseudocapacitors [3,4] according to their energy storage mechanism. Specifically, the energy storage of EDLCs is based on charge accumulation at the double-layer interfaces between electrolyte and electrode [5]. Therefore, the specific capacitance of an electrode is greatly affected by its specific surface area and pore structure [6]. While the energy storage of pseudocapacitors is based on the fast and reversible redox reaction at the surface of electrodes.

The electrode material is the most important factor for supercapacitors, which govern the electrochemical performance such as specific capacitance of supercapacitors. Generally, the electrode materials of supercapacitors are of three types, which are carbon-based materials [7,8], conducting polymers [9,10,11], and transition metal oxides [12,13,14]. Among these three types of electrode materials, carbon-based materials are the most widely used for electrode manufacturing. Various carbon-based electrode materials have been made and evaluated for the production of electrical EDLCs such as activated carbons, carbon nanotubes [15], carbon aerogels [16], graphene sheets [17,18], and carbon nanofibers [19]. Among these electrode materials, activated carbon electrodes are the most commonly used because of cost-effectiveness and high cycling durability [20]. 

Recently, the electrospun carbon nanofibers have been reported as ideal materials for fabrication of binder-free electrodes with excellent performance [21,22]. Electrospinning is a well-known fiber production technique that could fabricate continuous nanofibers with uniform diameters in the sub-micrometer to few nanometers and large surface area. After stabilization and carbonization, the as-spun nanofibers can be converted to carbon nanofibers. Polyacrylonitrile (PAN) is the most common-used polymer precursor for preparation of carbon nanofibers through electrospinning and thereafter stabilization and carbonization processes. Despite the carbon nanofibers from PAN have superior properties such as high mechanical strength and surface area, the use of petroleum-based PAN can be problematic due to the depletion of fossil fuel resources. Moreover, the high cost of PAN restrains its large scale applications. Therefore, there is an increasing globe interest for developing and utilizing renewable and inexpensive resources for carbon nanofiber fabrication 

Lignin, the second most abundant biopolymer on Earth, is available in large quantities from wood-pulping industries [23,24]. The commercially available lignins from wood-pulping include kraft lignin and lignosulfonates have been insensitively studied for the preparation of activated carbon, carbon-based nanomaterials, resins, and polymers [25]. In recent years, enzymatic hydrolysis lignin (EHL), which is a new type of lignin extracted from biomass enzymatic hydrolysis process during bio-ethanol production, has drawn a strong global interest both in academia and industry due to the growing number of bio-ethanol industries and increasing output of EHL. EHL is mainly treated as a waste and is usually burned onsite for heat and power regeneration. This results in enormous environment pollutions and the waste of the lignin resource [26]. Since EHL is a kind of polyaromatic macromolecule with a carbon content of 55–66%, it can be potentially used as an alternative of PAN for the production of carbon nanofibers. Hence, to the best of our knowledge, there is no research to fabricate carbon nanofibers used EHL as a precursor.In the present study, we evaluated the use of EHL as a polymer blend for the preparation of carbon nanofibers through an electrospinning technique and followed by stabilization and carbonization. An electrode was fabricated by the carbon nanofibers. Additionally, we applied a simple electrode preparation method for supercapacitor application. Specifically, in a transitional electrode preparation process, the carbon materials are usually ground to fine powders followed by glue the powders onto nickel foams to produce an electrode. In our simple electrode preparation process, the lignin-based electrospun CNFs (which is actually a membrane) is directly cut and crushed into a sheet as an electrode in Figure S1 (in Supplymentry Materials). The morphological and structural properties ECNF membranes were characterized by scanning electron microscopy (SEM), Raman spectra, X-ray photoelectron spectroscopy (XPS), and nitrogen sorption measurements, respectively. The electrochemical performances of carbon nanofiber membranes were further studied by cyclic voltammetry (CV), galvanostatic charge/discharge, and electrochemical impedance spectroscopy (EIS).

## 2. Materials and Methods

### 2.1. Materials

The EHL powder was purchased from LongLive Inc. (Shandong province, Jinan, China). Poly(acrylonitrile) (PAN) (*M*_w_~150,000) was purchased from Sigma-Aldrich Inc., St. Louis, MO, USA. *N*,*N*-dimethylformamide (DMF) was purchased from Nanjing Chemical Reagent Inc, Nanjing, China.

### 2.2. Experimental

#### 2.2.1. Preparation of ECNFs

**Figure 1:**
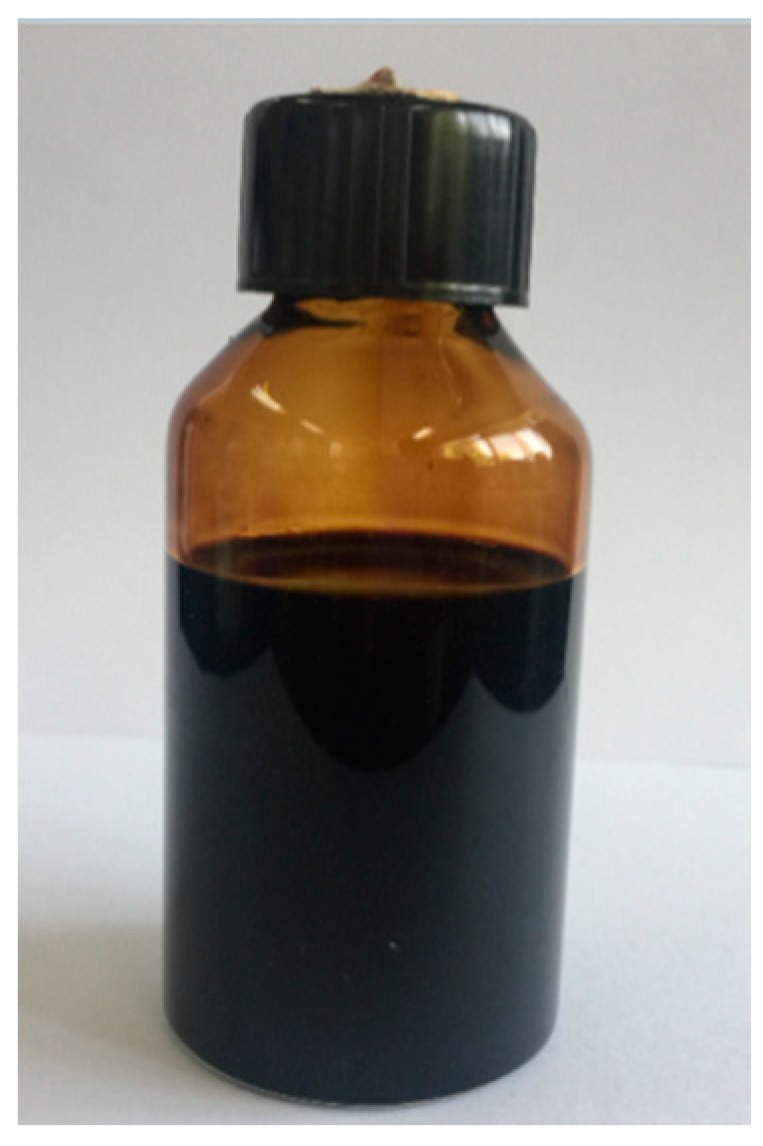
Homogeneous PAN/EHL composite solution.

The spin dopes with PAN/EHL mass ratios of 100/0, 60/40, 40/60, and 30/70 were prepared in DMF with the concentration of solutions was 12%. Specifically, the PAN was first dispersed in an appropriate amount of DMF, followed by the addition of corresponding amounts of EHL into the solutions at 80 °C under constant stirring to avoid agglomeration of the lignin particles. Figure 1 shows the as-prepared homogeneous PAN/EHL solution for electrospinning.

The composite solutions were then loaded into a 5-mL plastic, disposable syringe with a 22-gauge needle. The needle was connected to the positive terminal of a voltage generator. An operating voltage of 25 kV and a flow rate of 4.8 mL/h were applied in electrospinning experiments. An aluminum plate of 15 cm diameter covered with a thin aluminum foil, which was connected to the negative electrode of power supply used as a collector. The working distance was adjusted to 25 cm. Electrospinning was performed at room temperature and at a relative humidity between 30% and 40%. After dried under vacuum at 60 °C for 24 h, the collected electrospun fibers were obtained and kept in a desiccator.

**Scheme 1:**
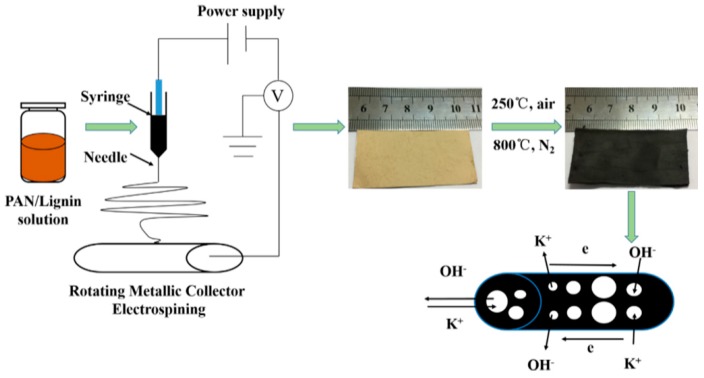
Schematic illustrating the fabrication steps and ion/electron transport behavior in ECNFs electrode.

To convert lignin/PAN composite nanofibers to carbon nanofibers, stabilization, and carbonization are required. For carbonization, the composite nanofibers were treated at 250 °C for 90 min under an air atmosphere at a heating rate of 1 °C/min. After thermal stabilization, the samples were subsequently placed into a tube furnace with a flow of N_2_ and carbonized at 800 °C for 60 min at a heating rate of 10 °C/min. The fabricated carbon nanofiber membranes were denoted as ECNFs-0, ECNFs-4, ECNFs-6, ECNFs-7 according to the amount of lignin. The flow diagram of lignin-based carbon nanofibers synthesis process is illustrated in Scheme 1.

#### 2.2.2. Characterization of Lignin-Based Carbon Nanofibers

The morphology of nanofibers was examined using a field emission scanning electron microscope (FE-SEM, JSM-7600F, Tokyo, Japan) operating at 30 kV. A patch of the samples was fixed on carbon tape and the sputtered with Au/Pd. The average fiber diameter of each sample was determined by measuring diameters of 50 randomly selected nanofibers in the corresponding FE-SEM image using the ImageJ software (Bethesda, MD, USA). Nitrogen adsorption measurement was performed at 77 K on a micrometric ASAP 2420 analyzer (Norcross, GA, USA). The specific surface area (SSA) of the samples was calculated by the Brunauer-Emmett-Teller (BET) method. The pore size distribution (PSD) was summarized by density functional theory (NLDFT) model. X-ray photoelectron spectroscopy (XPS) spectra were collected on an AXIS ULTRA spectrometer (Kratos Analytical, Tokyo, Japan) using a monochromatized Al-Kα X-ray source. Raman spectra were measured on a DXR532 Raman spectrometer (Themor, Waltham, MA, USA) with an excitation wavelength of 532 nm.

#### 2.2.3. Electrochemical Measurements

A three-electrode system was assembled to characteristic the electrochemical performance of the ECNFs by cyclic voltammetry (CV), galvanostatic charge-discharge (GCD), and electrochemical impedance spectroscopy (EIS) on a Gamary Reference 3000 electrochemical workstation (Warminster, PA, USA) with 6.0 M KOH as the aqueous electrolyte. The actual 3-electrode setup was showed in Figure S2. In general, organic or polymeric materials are required as binder for preparing activated carbons based electorde; whereas binders would have negative effects on the performance of electrode [27]. So the carbon nanofibers were cut into a square with the surface area of 1 × 1 cm^2^. Then the electrode sheet was pressed onto nickel foam with a pressure of 20 MPa to ensure a good contact. 

## 3. Results and Discussion

### 3.1. Morphology

**Figure 2:**
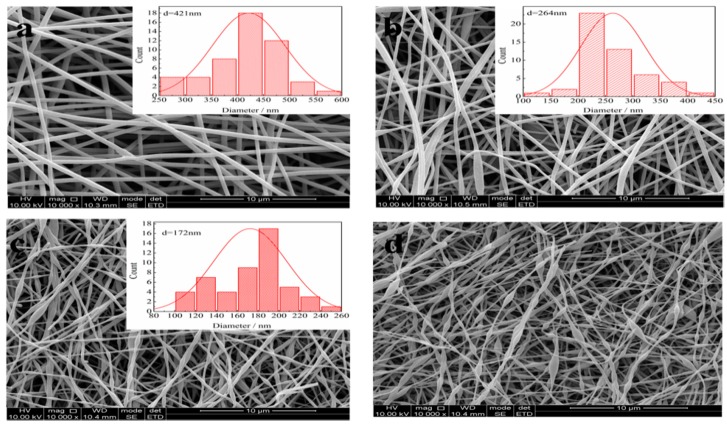
SEM images of different ECNF membranes: (**a**) ECNFs-0; (**b**) ECNFs-4; (**c**) ECNFs-6; and (**d**) ECNFs-7.

Due to lack of chain structures or molecular entanglements, an aqueous mixture containing lignin alone could not be electrospun into nanofibers [27]. However, with the combination of PAN, uniform lignin-based carbon nanofibers can be readily obtained. Figure 2 shows the SEM images of as-synthesized lignin-based nanofibers with different EHL contents. The diameter distributions of the carbon nanofibers were also shown in the inserted images in Figure 2. SEM results revealed that the diameter of nanofibers was strongly influenced by the EHL content in the spinning solution. The average diameter of carbon nanofibers decreased from 421 to 172 nm with the increased of the EHL amount from 0 to 60 wt. % (Figure 2a–c). Further increased in the EHL content to 70 wt. % resulted in a dramatic change in nanofiber morphology, where the diameter of nanofiber became heterogeneous and nanofiber skeleton was filled with abundant beads (Figure 2d). This morphology changes could be attributed to the decreased viscosity of the spin dope when PAN was gradually replaced by low molecular weight EHL [27]. The low molecular of lignin could not form the effective chain entanglements in the dilute electrospinning solution jets, as a result, as-spun nanofibers tend to break or have heterogeneous diameters when the stretch force is applied on the jets during electrospinning [28]. Moreover, the viscoelasticity of electrospinning jets, which facilitates the shrinkage of as-spun fibers and led to the formation of bead nanofibers [29]. 

### 3.2. Chemical Properties and Pore Structure

**Figure 3:**
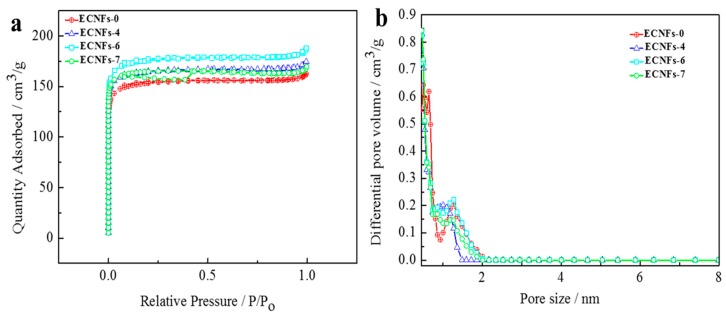
(**a**) N_2_ adsorption-desorption isotherms; and (**b**) pore size distribution of ECNFs.

To further determine the structure of the ECNFs, the BET specific surface area and pore size distribution (PSD) were tested using N_2_ adsorption-desorption and the results shown in Figure 3. It could be seen from Figure 3a that all ECNFs display significantly enhanced N_2_ uptake and obvious capillary condensation at relative pressure (P/P0) below 0.1, which was due to the existence of abundant micropores between nanofibers which lead to type I adsorption of N_2_. The pore size distribution (PSD) was shown in Figure 2b. As could be seen, all the samples have PSD peaks between 0.2–2 nm (Table 1), indicating the formation of micropores during the carbonization. The specific surface area and pore size distribution are two critical factors for the electrochemical performance of carbon nanofibers when used as a supercapacitor. The ideal carbon electrodes are required to have a high surface area and an appropriate pore size distribution. The macropores provide high buffer volume for electrolyte ions, the mesopores acting as channels for the fast transportation of ions, and the micropores can enhance the ion-accessible surface area for the construction of electrical double layer. Table 1 summarized the pore structure parameters of ECNFs. As the increase of lignin content in nanofibers from 0 to 60%, the BET surface area was increased from 578 to 675 m^2^/g, while the pore volumes for both micro- and mesopores was not changed much. It is worth to note that the of such a high surface area ECNFs prepared without an activation process have been not reported before. When ions get through macropores, ions could enter into micropores quickly due to deficient mesopores. These microspores could provide ECNFs with fast ion transport channels and large accessible surface area for the construction of electrical double layer. The ECNFs could take full advantage of the microspores to generate outstanding electrochemical performances.

**Figure 4:**
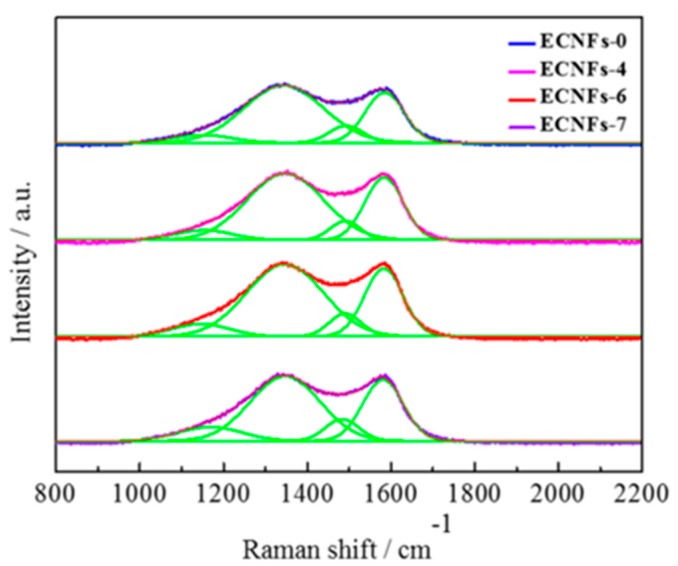
Raman spectra of ECNFs.

Figure 4 shows the Raman spectra of ECNFs. Two peaks located at 1340 and 1593 cm^−1^ in Raman spectra can be attributed to the D and G bands of carbon. D band was related to the disordered sp^3^ carbon atoms, whereas G band at 1593 cm^−1^ corresponded to sp^2^-hybridized carbon atoms in the graphitic layers [30]. The D band can be further resolved into three pseudo-Voigt shaped peaks centered at 1170, 1340, and 1500, attributed to D4, D1, and D3. The D3 is related to amorphous carbon [31,32]. The D4 band was related to ions impurities, i.e., lignin ash and interstitial defects [33]. The ratio of intensities D1 and G (I_D__1_/I_G_) represents the degree of structural disorder of carbon materials. The I_D__1_/I_G_ ratios of ECNFs were calculated to be 0.87, 0.94, 0.93, and 0.93, respectively. Compared with reference carbon fibers (ECNFs-0), lignin-based ECNFs have higher I_D__1_/I_G_ values suggested their higher degree of structural disorder. This is because the introduction of lignin into PAN generated many structural defects in ECNFs, as a result, increased the surface area and total pore volume of ECNFs. Moreover, lignin is a kind of heterogeneous macromolecular compound that has complex thermal decomposition behavior [34]. Thereby, carbonization of lignin would form a more disordered carbon structure than that of PAN. In addition, Raman spectroscopy only gives the surface structure at ~10 nm depth, it is possible that the interfacial region between lignin-derived carbon and PAN carbon nanofibers could be slightly more graphitic [35].

**Figure 5:**
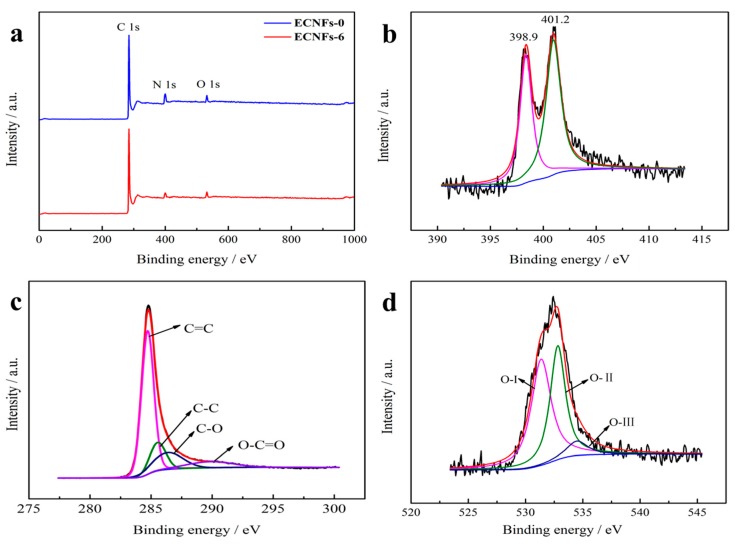
(**a**) XPS spectrums of ECNFs-0 and ECNFs-6; (**b**) C 1s peaks of ECNFs-6; (**c**) N 1s peaks of ECNFs-6; and (**d**) O 1s peaks of ECNFs-6.

The above characterization results suggested that ECNFs-6 has the smallest average fiber diameters and largest surface and pore volumes among all as-prepared lignin-based ECNFs. Thus, the ECNFs-6 was selected for further characterization using XPS. The XPS spectra of ECNFs-0 and ECNFs-6 show similar patterns, both contained high C 1s (285 eV), faint O 1s (532 eV), and N 1s (400 eV) peaks. In comparison with ECNFs-0, ECNFs-6 contained more carbon but less nitrogen and oxygen (Table 2), this can be attributed to the substitution of PAN to nitrogen free lignin compound. The C 1s peak can be deconvoluted to four individual peaks at approximately 284.8, 285.7, 286.5, and 290 eV (Figure 5b), which can be attributed to sp^2^ C=C, sp^3^ C–C, C–O, and O–C=O bands, respectively [36]. Therefore, there are some oxygen-contained functional groups in the carbonization procedure. Moreover, the N 1s peaks can be deconvoluted to two peaks located at binding energy of 398.9 and 401.2 eV (Figure 5c), which were attributed to pyridinic-N and quaternary-N, respectively [37]. According to reports, the introduction of the N could lead to great pseudocapacitance effect, improving the capacitance perform as a result [38]. Figure 3d shows the deconvoluted peaks of O 1s, which were corresponded to quinone groups or keto oxygen (O-I, 531.5 eV), ether groups or phenol groups (O-II, 532.9 eV), and water or carboxylic groups (O-III, 534.5 eV) [39]. These oxygen-related (especially O-I) functional groups along with nitrogen functional groups could provide extra pseudocapacitance to improve the specific capacitance [40].

### 3.3. Electrochemical Performance

**Figure 6:**
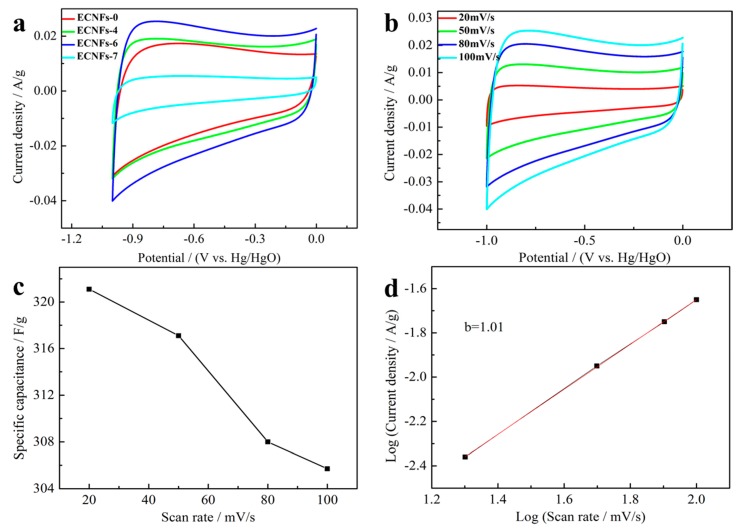
(**a**) CV curves at 100 mV/s; (**b**) CV curves of ECNFs-6 at different scan rates; (**c**) Rate performance of ECNFs-6; and (**d**) b-value was determined by fitting a log (discharge current density)–log (scan rate) plot.

The CV curves of the ECNFs electrodes were performed with the potential range of −1 to 0 V at the scan rate of 100 mV/s in 6 M KOH aqueous electrolyte, and the CV curves are shown in Figure 6a. The shape of the CV curves of ECNF electrodes can be classified to the quasi-rectangular type, suggesting typical double capacitor behavior [41]. Generally, the specific capacitance of an electrode is proportion to the integrated area of its CV profile under the same scan rate and voltage window [42]. The capacitance was calculated from CV curves according to the following equation:

(1) $$C_{\mathrm{CV}}=\frac{1}{\mathrm{vm}\Delta u}\int \mathrm{idu},$$

where i is the response current, v is the potential scan rate, u is the potential window, m is the mass of active materials on the single electrode.

The calculation results suggest that the capacitances of the EVNFs electrodes with different lignin content are as follows: ECNFs-6 (305.7 F/g) > ECNFs-4 (278.3 F/g) > ECNFs-0 (183.1 F/g) > ECNFs-7 (151.7 F/g). Generally, lignin-based ECNFs containing 40-60% of lignin greatly increased the electrode capacitance by 67% and 52% for ECNFs-6 and ECNFs-4, respectively. This enhancement is attributed to lignin-based ECNFs have higher surface areas and pore volumes. Among the samples, the ECNFs-6 shows the highest specific capacitance due to its highest specific surface area. In addition, these curves also exhibit broadened peaks at −0.8 to −0.2 V, suggesting the pseudo-capacitance caused by the surface oxygen-containing groups [40], which were consistent with XPS data. However, the ECNFs-7 displayed the lower electrode capacitances in spite of it has a higher SSA than that of ECNFs-0. This phenomenon may be attributed to the higher amount micropores in ECNFs-7 are actually not accessible for K+ and/or OH− during the charge/discharge process although those micropores did contribute to a high SSA. In addition, ECNFs-7 was mainly consisted of heterogeneous bead fibers which hindered the diffusion of ions during charging/discharge process. The CV curves of ECNFs-6 at different scan rates (20, 50, 80, and 100 mV/s) were further studied shown in Figure 6b. With the increase of scan rate, the CV curves still keep the quasi-rectangular shape, indicating a reversible and stable supercapacitor behavior. When scan rate increased from 20 to 100 mV/s, the capacitance retention of ECNFs-6 was from 321.1to 305.7 F/g (Figure 4c), prior to that of other porous carbons [43,44], exhibiting a good rate performance. 

It has been reported that the charge storage mechanism can be evaluated by the following equation:

(2) $$i=av^{b}$$

where *i* is the peak current (mA); v is the scan rate (mV/s); *a* and *b* are coefficients. The value *b* can be used to evaluate the charge storage whether it caused by capacitive behavior or diffusion-controlled processes, in which the *b* = 0.5 indicates a semi-infinite diffusion-controlled process, while *b* = 1 represents a capacitive behavior. The ECNFs-6 was selected in this study to reveal the charge storage mechanism of lignin-based ECNFs. The discharge current densities at −0.5 V were extracted from the CV curves and plotted the log (discharge current density (A/g)) vs. log (scan rate (mV/s)) in Figure 4d. By fitting the plot, the coefficient *b* = 1.01 was obtained. The result suggests that energy storage in the ECNFs-6 device was achieved by fast and reversible electrostatic ion adsorption at the electrode/electrolyte interface [45], and indicates that a surface-controlled capacitive electrode process dominates the ECNFs-6 nanochannels electrode [46].

**Figure 7:**
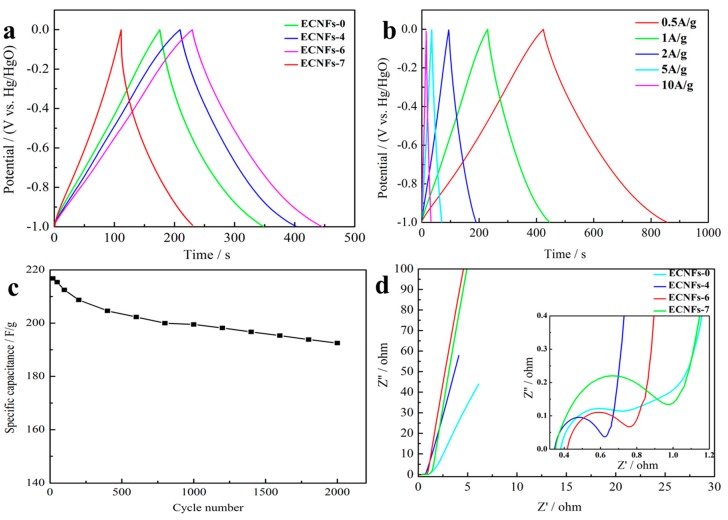
(**a**) GCD curves at 1 A/g; (**b**) GCD curves of ECNFs-6 at different current density; (**c**) Cycling performance ECNFs-6 at acurrent density of 1 A/g; and (**d**) Nyquist plots of ECNFs-based electrode with the high-frequency region and equivalent circuit diagram given in the inset.

GCD tests were also measured to reveal the electrochemical properties of the ECNF electrodes, with the results shown in Figure 7a. These curves showed regular triangular shapes between −1 to 0 V, indicating outstanding coulombic efficiency and reversibility [41], which confirms that the double-layer capacitance dominates the total capacitances [40]. Furthermore, the specific capacitance was calculated from the GCD curves according to the following equation:

(3) $$C_{\mathrm{GCD}}=\frac{I\Delta t}{m\Delta u}$$

*u*, *t*, *m*, and *I* have the same meaning in Equation (1).

The specific gravimetric capacitance values of ECNFs-0, ECNFs-4, ECNFs-6, and ECNFs-7 were 173.5, 194.4, 216.8, and 119.5 F/g, respectively, whose tend was consist with the CV results. The GCD curves of the ECNFs-6 electrode under different current densities (Figure 7b) kept a triangular shape, suggesting that the electrode could be applied in the areas which fast ion transportation and high current density are required [27]. Figure 7c shows cycling performance ECNFs-6 at a current density of 1 A/g. It could be observed that ECNFs-6 retained high cycling stability of 92% capacitance retention 1000 cycles at 1 A/g. The excellent capacitive performances of ECNFs-6 can be attributed to the following aspects: (a) numerous macropores act as the ion reservoir and shorten the ion diffusion distance to the interior carbon nanofiber surfaces, (b) abundant micropores could observably increase the specific surface area which can improve the electric double-layer capacitance of electrodes, (c) oxygen (especially O-I) and nitrogen functional groups provide extra pseudocapacitance.

Figure 7d shows the Nyquist plot of the samples measured in 6 M KOH solution. In the low-frequency region, the straight line was nearly parallel to the imaginary axis, revealing the electrical double layer capacitive behaviors [47]. The ohmic resistance is acquired from the first intercept value in the real axis, which contains the resistance of the electrolyte, current collector, active material, coin cells, separator, and the contact resistance of the current collector with the active materials [48]. The ECNFs present low ohmic values of 0.38, 0.34, 0.41, and 0.35 Ω, respectively, which corresponded to the equivalent series resistance (Rs). High equivalent series resistance would lead to low electrical conductivity, which would further deteriorate the performance of supercapacitor. Different diameters of the semicircle indicated that the ion diffusion efficiency. ECNFs-7 has the maximum charge transfer resistance due to its maximum semicirlce diameter. As a result, the capacitance of ECNFs-7 was minimum. The rather low Rs implies a good ion conductivity and a fast electron transfer process of the device. 

**Figure 8:**
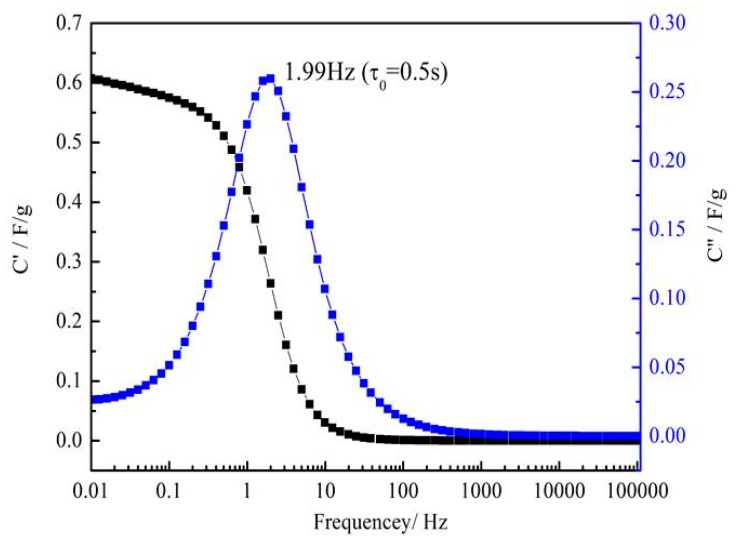
Real (C′) and imaginary (C′′) parts of specific capacitance of ECNFs-6.

Bode plot of the ECNFs-6 is shown in Figure 8a. The imaginary part of capacitance ECNFs-6 goes through a maximum at a frequency f_0_ (1.98 Hz), which defines the relaxation time constant τ_0_ (= 1/f_0_, the minimum time for all the energy to be discharged from the supercapacitor with an efficiency >50% [40]) to be 0.5s. This value was comparable with carbon nanomesh [49] and holey graphene foam electrode [50] (0.46 and 0.49 s), but was better than activated carbon electrode (1.2 [51] and 2.45 [30] s). It is demonstrated that the feasibility of ECNFs-6 as a supercapacitor electrode.

## 4. Conclusions

Lignin-based ECNFs were prepared by electrospinning EHL/PAN hybrid solution into precursor nanofibers, followed by stabilization in air and carbonization in N_2_. The characterization results indicated that ECNFs with as high as 60% lignin could retain homogeneous fiber morphology and high surface area. Moreover, ECNFs were used as supercapacitor electrodes without the addition of any binder and the electrochemical performances were studied by CV, GCDand EIS. ECNFs containing up to 60% of lignin presented excellent performance as supercapacitors, i.e., high capacitance (up to 216.8 F/g), good cyclic life (88.8% capacitance retention after 2000 cycles), small time constant (0.5 s). It is envisioned that the ECNFs made from EHL could be innovative and sustainable electrode materials for high-performance supercapacitors.

## Figures and Tables

**Table 1:** Pore structure parameters of ECNFs.

Samples	SBET/m^2^/g	Smicro/m^2^/g	Vmicro/cm^3^/g	Vmeso/cm^3^/g	Vtotal/cm^3^/g
ECNFs-0	578	523	0.21	0.04	0.25
ECNFs-4	635	589	0.24	0.02	0.26
ECNFs-6	675	607	0.24	0.05	0.29
ECNFs-7	631	571	0.23	0.04	0.27

SBET, specific surface area; Smicro, specific surface area of micropores; Vmicro, the micropores volume; Vmeso, the mesopores volume; Vtotal, the total pore volume.

**Table 2:** The element compositions of ECNFs-0 and ECNFs-6 from XPS analysis.

Samples	C%	N%	O%
ECNFs-0	86.57	10.04	3.4
ECNFs-6	91.02	5.69	3.29

## Supplementary Materials

The supplementary materials are available online at http://www.mdpi.com/2073-4360/10/12/1306/s1.
