# Supplementary material for: Electrospun Enzymatic Hydrolysis Lignin-Based Carbon Nanofibers as Binder-Free Supercapacitor Electrodes with High Performance
Source: Polymers (Basel). 2018 Nov 26;10(12):1306. doi: 10.3390/polym10121306 (PMC6401840; doi:10.3390/polym10121306)
Supplement: Supplementary file 1 [file polymers-10-01306-s001.pdf]

Supporting Information

Electrospun Enzymatic Hydrolysis Lignin-Based Carbon Nanofibers as Binder-Free Supercapacitor Electrodes with High Performance

Xiang Wang1, Wei Zhang[1], Minzhi Chen[1], Xiaoyan Zhou[1,*]

1 College of Wood Science &Technology, Nanjing Forestry University, 159, Longpan Road, Nanjing, Jiangsu Province, P.R. China;

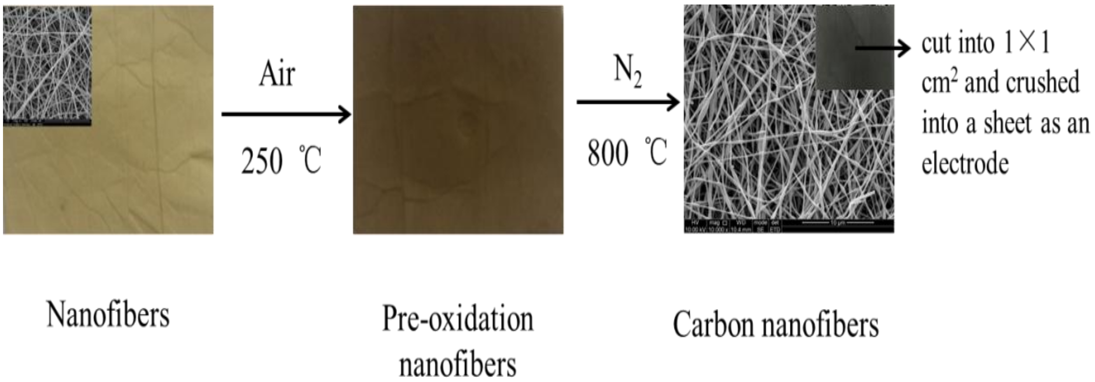

Figure S1. The fabrication steps of an electrode.

In this manuscript, we applied a simple electrode preparation method for supercapacitor application. Specifically, in a transitional electrode preparation process, the materials are usually ground to fine powders followed by glue the powders onto nickel foams to produce an electrode. In our simple electrode preparation process, the lignin-based electrospun CNFs (which is actually a membrane) is directly cut and crushed into a sheet as an electrode.

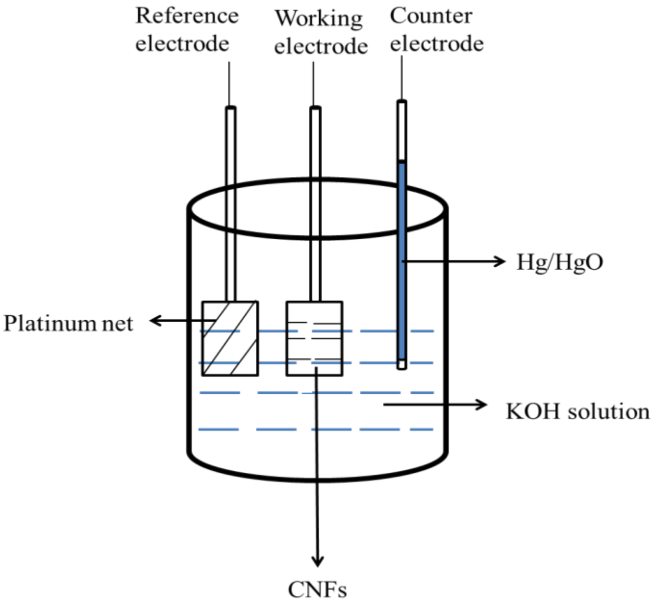

Figure S2. The actual three-electrode setup.

The reference electrode is used for ensure the potential of working electrode. The counter electrode is used for conducting current. Before electrochemical measurements, the working electrode will be pretreated by vacuum impregnation in KOH solution in order to fully infiltrate the working electrode.
